# Fibro-adipose vascular anomaly (FAVA): three case reports with an emphasis on the mammalian target of rapamycin (mTOR) pathway

**DOI:** 10.1186/s13000-020-01004-z

**Published:** 2020-07-25

**Authors:** Yumiko Hori, Katsutoshi Hirose, Noriko Aramaki-Hattori, Sachi Suzuki, Robert Nakayama, Masanori Inoue, Takahiro Matsui, Masaharu Kohara, Satoru Toyosawa, Eiichi Morii

**Affiliations:** 1 Department of Pathology, Osaka University Graduate School of Medicine, 2-2 Yamada-oka, Suita, Osaka, 565-0871 Japan; 2 Department of Oral Pathology, Osaka University Graduate School of Dentistry, 1-8 Yamada-oka, Suita, Osaka, 565-0871 Japan; 3 Department of Plastic and Reconstructive Surgery, Keio University School of Medicine, 35 Shinanomachi, Shinjuku-ku, Tokyo, 160–8582 Japan; 4 Department of Plastic and Reconstructive Surgery, Japanese Red Cross Shizuoka Hospital, 8-2 Outemachi, Aoi-ku, Shizuoka-shi, Shizuoka, 420-0853 Japan; 5 Department of Orthopaedic Surgery, Keio University School of Medicine, 35 Shinanomachi, Shinjuku-ku, Tokyo, 160–8582 Japan; 6 Department of Diagnostic Radiology, Keio University School of Medicine, 35 Shinanomachi, Shinjuku-ku, Tokyo, 160–8582 Japan

**Keywords:** Fibro-adipose vascular anomaly (FAVA), PIK3CA, mTOR, Vascular anomaly

## Abstract

**Background:**

Fibro-adipose vascular anomaly (FAVA) is a new entity of vascular anomalies with somatic and mosaic gain-of-function mutations of the *phosphatidylinositol-4, 5-bisphosphate 3-kinase catalytic subunit alpha* (*PIK3CA*). *PIK3CA* mutation excessively activates mammalian target of rapamycin (mTOR) pathway, which promotes angiogenesis and lymphangiogenesis. Histologically, FAVA is composed of intramuscular fibrous and adipose tissues with venous malformation (VM). Although sirolimus known as a mTOR inhibitor has good response to FAVA, expression pattern of the mTOR pathway was still unclear. Herein, we immunohistochemically investigated three novel FAVA patients with an emphasis on the mTOR pathway (p-S6K1, p-4EBP1 and p-AKT).

**Case presentation:**

Case 1: A 10-year-old female had complained of pain in the left thigh since she was 6-year-old. Under the clinical diagnosis of VM, she underwent surgical resection for the lesion. Case 2: A 29-year-old female patient had complained of discomfort and mild pain in the left shoulder since she was 18-year-old. After childbirth, she had severe ongoing pain and contracture of the shoulder. Under clinical diagnosis of VM, surgical resection was performed. Case 3: A 53-year-old female had complained of pain and knee restriction after surgical treatment of a knee tumor at the age of 31. Under the clinical diagnosis of atypical lipomatous tumor or high grade liposarcoma, surgical resection was performed. Histologically, all three patients presented with characteristic features of fibrous and adipose tissues with abnormal vessels within the skeletal muscle, leading to diagnosis of FAVA. Although VM has been reported as an important finding in FAVA, immunohistological findings demonstrated that abnormal vessels comprised complex of VM and lymphatic malformation (LM) in all cases. Furthermore, besides vascular malformation, abnormal fibrous and adipose tissues of FAVA expressed mTOR pathway components.

**Conclusions:**

We presented three new cases of FAVA. Histological and immunohistochemical analyses revealed that VM and LM complex was an important finding in FAVA, and that the mTOR pathway components were expressed in abnormal fibrous tissue, adipose tissue and vascular malformation. These findings suggested that FAVA might be a mesenchymal malformation caused by PI3K/AKT/mTOR pathway.

## Background

Fibro-adipose vascular anomaly (FAVA) is a new entity of vascular anomalies, and has not been included in the International Society for the Study of Vascular Anomalies (ISSVA) classification [1]. FAVA is an extremely rare and a recently described vascular anomaly with approximate 20 cases in the literatures [1–5]. It is often present during young age, and occurs in the muscle of the lower extremities (about 90%) followed by the upper extremities and trunk. The common presenting symptoms are pain (100%), functional restriction (81%) and swelling (62%) [2]. FAVA affects females more frequently than males in a ratio of 3:1 [3, 4]. Histologically, FAVA is a complex mesenchymal malformation characterized by venous malformation (VM) surrounded by focal or diffuse fibro-adipose tissue within the skeletal muscle [1]. However, the abnormal lymphatic vessels accompanying VM were present in a few cases [1, 4]. Although VM was an important finding in FAVA, there was some room for discussion of the type of vascular malformation in FAVA.

Recent study identified that somatic and mosaic gain-of-function mutations of the *phosphatidylinositol-4,5-bisphosphate 3-kinase Catalytic Subunit Alpha* (*PIK3CA*) gene were found in FAVA, and FAVA belongs to the spectrum of PIK3CA-related overgrowth syndromes (PROS) [5–7]. PIK3CA encodes the 110-kD catalytic α-subunit of PI3K, which is the key lipid kinase that controls signaling pathways involved in cell proliferation, motility, survival and metabolism [8]. Activation of PI3K via different receptors results in phosphorylation of AKT, and p-AKT phosphorylates mammalian target of rapamycin (mTOR). Furthermore, phosphorylated form of mTOR also phosphorylates the downstream target of the ribosomal protein S6 kinase 1 (S6K1) and eukaryotic translation initiation factor 4E-binding protein 1 (4EBP1), which have a stimulatory function of increase in protein synthesis and cell growth in angiogenesis and lymphangiogenesis [7, 9–12]. In vitro study demonstrated that *PIK3CA* mutation excessively promoted activation of mTOR pathway [7, 13]. Moreover, sirolimus known as a mTOR inhibitor has an antiproliferative effect on various vascular anomalies including FAVA [3, 14, 15]. Considering these findings, activated PI3K/AKT/mTOR signaling pathway plays a key role in pathogenesis of vascular anomaly in FAVA. In this study, we reported three novel cases of FAVA, in which the immunohistochemical profile of blood vessel, lymphatic vessel and mTOR pathway was investigated.

## Case presentation

### Clinical findings

#### Case 1

**Fig. 1:**
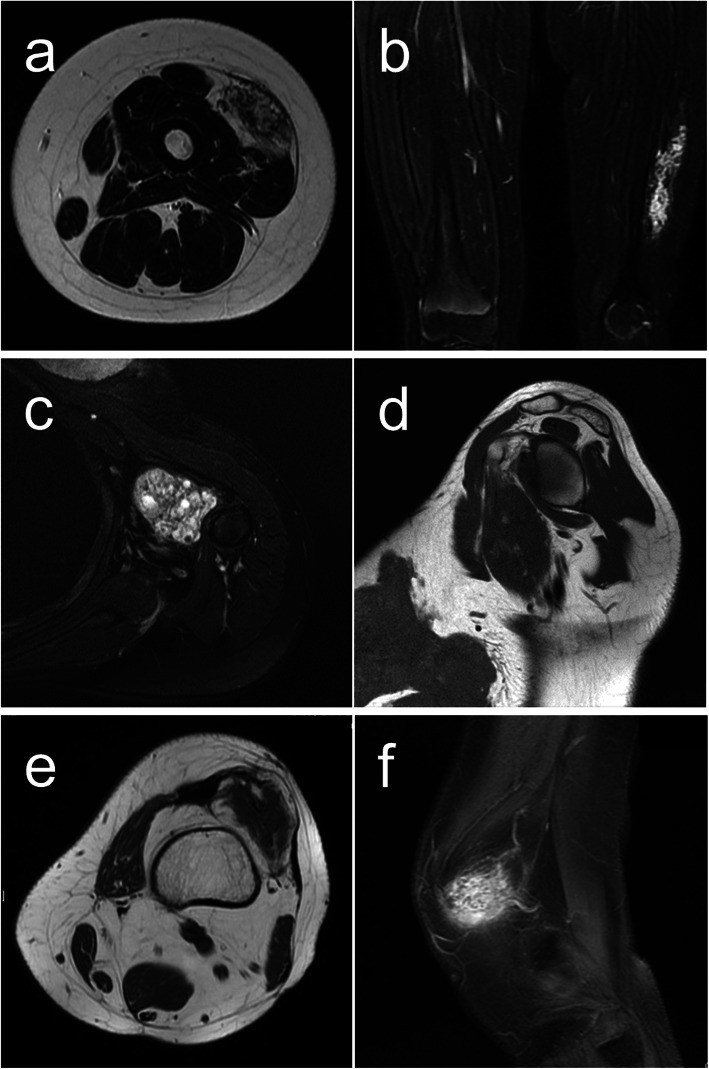
Magnetic resonance imaging (MRI). Axial T2-weighted MRI (**a**) and coronal fat-saturated enhanced T1-weighted MRI (**b**) of case 1. Axial fat-saturated T2-weighted MRI (**c**) and sagittal T1-weighted MRI (**d**) of case 2. Axial T1-weighted MRI (**e**) and sagittal fat-saturated T2-weighted MRI (**f**) of case 3

A 10-year-old female had complained of pain in the left thigh since she was 6-year-old. She had no significant prior medical or surgical history. Both axial T2-weighted Magnetic resonance imaging (MRI) and coronal fat-saturated enhanced T1-weighted MRI revealed heterogenous hyperintense within the skeletal muscle (Fig. 1a, b). The clinical diagnosis was VM. Restriction of the knee joint gradually appeared. Although sclerotherapy was attempted, no benefit was obtained. Additional ultrasonographic examination imaging showed an ill-defined hyperechoic intramuscular solid mass. Needle biopsy was performed, and VM was diagnosed. Then, surgical resection was performed.

#### Case 2

A 29-year-old female patient had complained of discomfort and mild pain in the left shoulder since she was 18-year-old. After childbirth, she had severe ongoing pain and contracture of the shoulder. She had no significant prior medical or surgical history. Axial fat-saturated T2-weighted MRI revealed high signal intensity and sagittal T1-weighted MRI revealed low signal intensity within the muscle of the shoulder. (Fig. 1c, d). Under clinical diagnosis of VM, the lesion was resected.

#### Case 3

A 53-year-old female had complained of pain and knee restriction after surgical treatment of a knee tumor at the age of 31. The details of surgical treatment were unclear. Axial T1-weighted MRI revealed low signal with fat component and sagittal fat-saturated T2-weighted MRI revealed high signal intensity (Fig. 1e, f). Under the clinical diagnosis of atypical lipomatous tumor or high grade liposarcoma, needle biopsy was performed, and the diagnosis of angiomatosis was made. Then, surgical resection was performed. After the resection, knee restriction disappeared.

### Histological findings

**Fig. 2:**
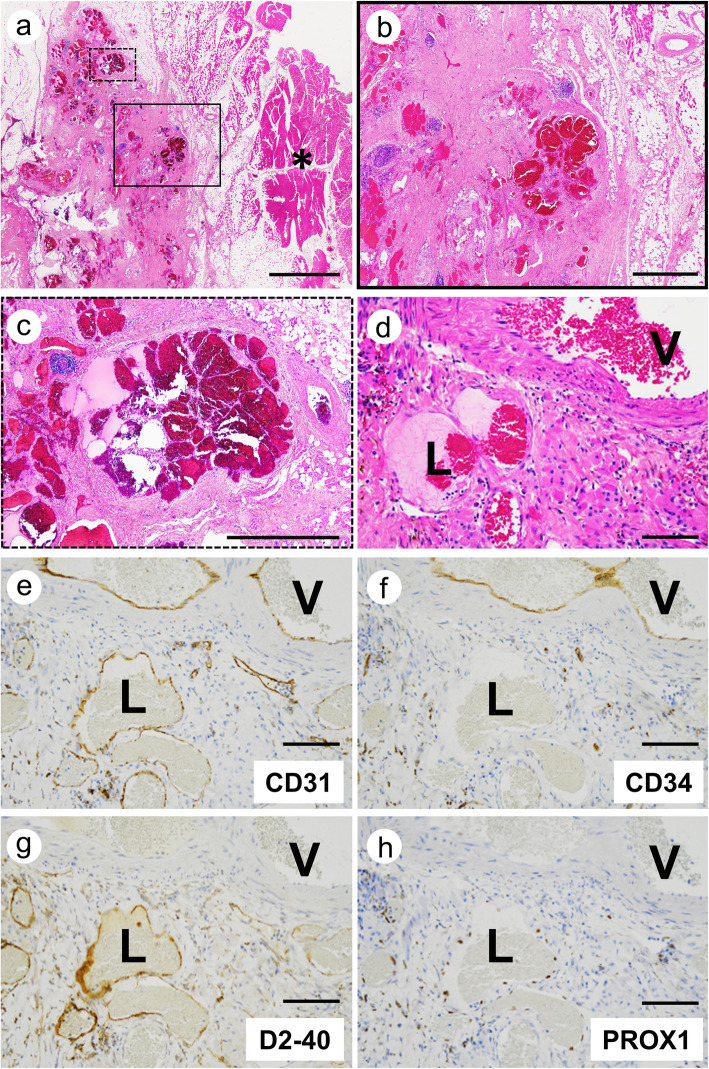
Histology and immunohistochemical analysis of vascular markers. Representative H&E staining of FAVA (**a**; loupe image, **b**; higher magnification of black box in a, **c**; higher magnification of dot box in a). Asterisk (*) indicated skeletal muscle surrounding FAVA lesion. Serial sections stained for H&E (**d**), CD31 (**e**), CD34 (**f**), D2–40 (**g**) and PROX1 (**h**). Abnormal veins (**V**) were positive for CD31 and CD34. Abnormal lymphatic vessels (**L**) were positive for CD31, D2–40 and PROX1. Scale bars: **a** = 5000 μm; **b, c** = 1000 μm; **d-h** = 100 μm

All three lesions were included in skeletal muscle (Fig. 2a). The most common findings were abnormal vessels and dispersed skeletal muscle islands surrounded by extensive fibrous and adipose tissue (Fig. 2b, black box in Fig. 2a). Moreover, the vascular clusters consisted of thin-walled back-to-back blood-filled sacs were observed (Fig. 2c, dot box in Fig. 2a). These clusters were frequently surrounded by lymphocytic aggregates (Fig. 2b, c). Although VM has been characterized as an important finding in FAVA, the abnormal vessels comprised complex of VM and lymphatic malformation (LM) in various proportions among our three cases (Fig. 2d). In case 1, LM accounted for a substantial fraction of abnormal vessels. VM component is large and irregular malformed vessels with muscularized walls. LM component is malformed microcysts lined by round endothelial cells. The other findings were organized thrombi within abnormal veins in two cases. Despite previous reports, no nerve containing enlarged venous vessels surrounded by dense fibrous tissue were seen in our cases [1].

### Immunohistochemistry

First, we performed CD31 (clone JC70A, 1:200, Dako), CD34 (clone QBEnd10, 1:200, Dako), D2–40 (clone dp36, 1:100, Dako) and PROX1 (ab199359, 1:500; Abcam, Cambridge, U.K.) immunostaining to provide additional evidence of VM and LM in FAVA. In abnormal veins, CD31 and CD34 were positive and D2–40 and PROX1 were negative in all three cases (Fig. 2e-h, **V**; abnormal veins). In contrast to abnormal veins’ staining, abnormal lymphatic vessels were positive for CD31, D2–40 and PROX1, and negative for CD34 (Fig. 2e-h, **L**; abnormal lymphatic vessels).

**Fig. 3:**
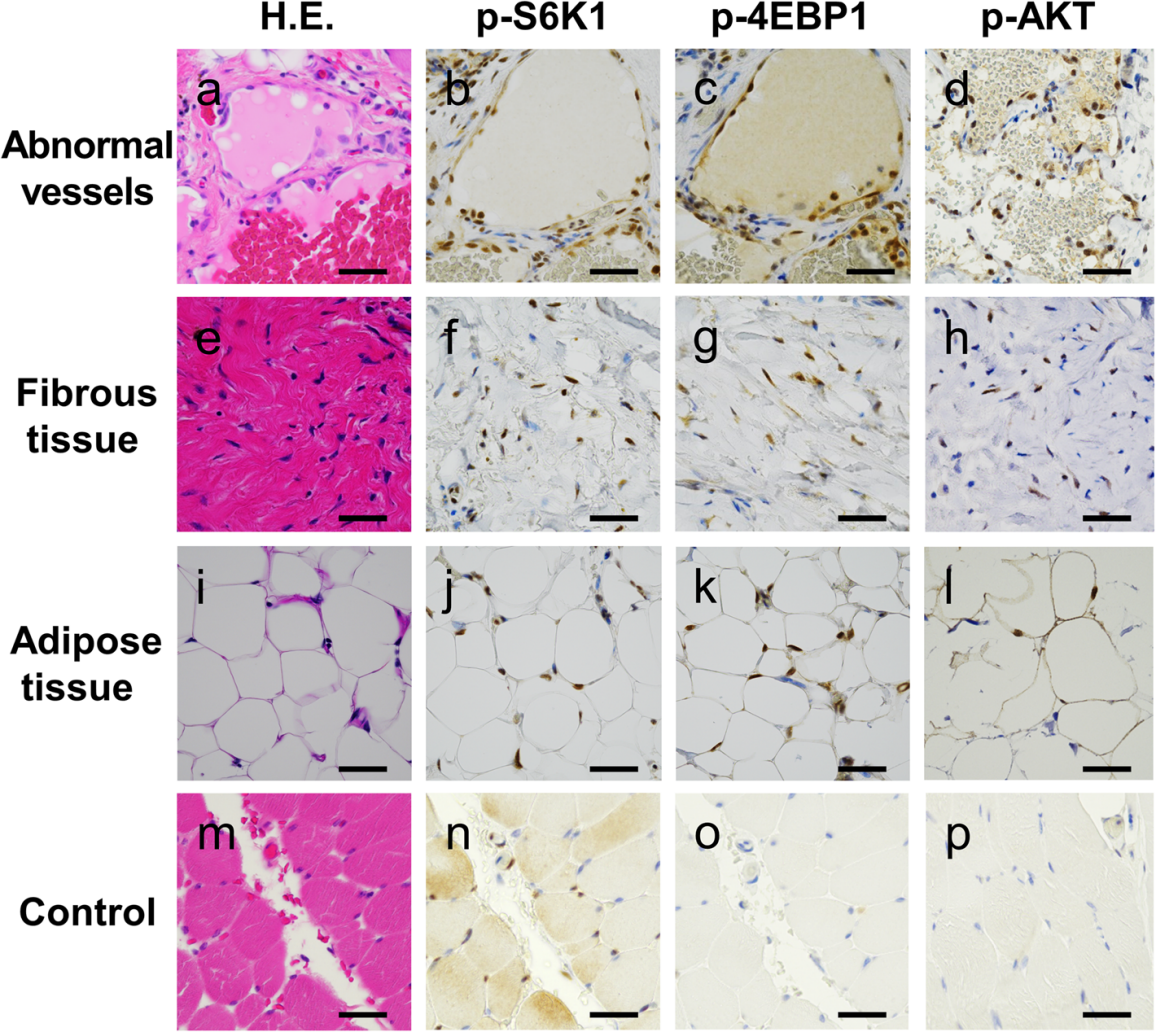
Immunohistochemical analysis of mTOR pathway in various components of FAVA. Abnormal vessels (**a-d**), fibrous tissue (**e-h**) and adipose tissue (**i-l**) in FAVA. Control (normal connective tissues surrounding FAVA) (**m-p**). Staining for H&E (**a, e, i, m**), p-S6K1 (**b, f, j, n**), p-4EBP1 (**c, g, k, o**) and p-AKT (**d, h, l, p**). Scale bars: **a-p** = 50 μm

Next, we analyzed the expression of mTOR pathway components, p-S6K1 (#9204, 1:100; Cell Signaling Technology, Danvers, MA, USA), p-4EBP1 (#2855, 1:500; Cell Signaling Technology) and p-AKT (#4060, 1:100; Cell Signaling Technology). Normal connective tissues surrounding FAVA lesions served as a control. Control normal tissues were mainly composed of skeletal muscle and normal vessels, because FAVA arises within skeletal muscle (Fig. 2a, asterisk). In our three cases, almost all abnormal vessels strongly expressed p-S6K1 (Fig. 3a, b) and the fibro-adipose tissue also expressed that (Fig. 3e, f, i, j). In addition, p-4EBP1 expression was detected in these FAVA components in all cases (Fig. 3c, g, k). The p-AKT expression was detected in abnormal vessels and fibrous tissue in all cases, besides sporadic p-AKT expression was detected in adipose tissue in two cases (Fig. 3d, h, l). On the other hand, p-S6K1 showed sporadic expression in skeletal muscle and normal vessels, whereas p-4EBP1 and p-AKT showed no expression in these tissues. (Fig. 3m-p). The summarized immunohistochemical results of all cases were shown in Table 1.

**Table 1:** Immunohistochemical expression of PI3K/AKT/mTOR pathway in various components

		p-S6K1	p-4EBP1	p-AKT
Case 1.	**Abnormal vessels**	+	+	+
	**Fibrous Tissue**	+	+	+
	**Adipose Tissue**	+	+	+
Case 2.	**Abnormal vessels**	+	+	+
	**Fibrous Tissue**	+	+	+
	**Adipose Tissue**	+	+	+
Case 3.	**Abnormal vessels**	+	+	+
	**Fibrous Tissue**	+	+	+
	**Adipose Tissue**	+	+	–
Control	**Skeletal muscle, Vessels**	+	–	–

Staining intensity (−; no expression / +; positive)

## Discussion

In this study, we described three cases of previously unreported immunohistochemical characteristics of FAVA. Histological and immunohistochemical analyses revealed that lymphatic-venous malformation (LVM) was an important finding in FAVA, and the mTOR signaling pathway was expressed in various FAVA components.

Identification of vascular type was conducted using CD34 as markers for blood vessels, as well as D2–40 and PROX1 for lymphatic vessels. Histological and immunohistological findings demonstrated presence of complex of abnormal veins and lymphatic vessels in all three cases (Fig. 2d-h). The proportion of abnormal veins and lymphatic vessels was varied among our cases. There is a possibility that vascular malformation composing only VM in previous cases may include LM component [1–5]. LVM is combined vascular malformation (VM + LM). ISSVA defines “combined vascular malformation” as two or more vascular malformations found in one lesion. Thus, in FAVA, vascular malformation component may be LVM rather than VM.

Immunohistochemistry demonstrated that abnormal vessels of all our cases expressed the p-AKT and mTOR effector, p-S6K1 and p-4EBP1 (Fig. 3a-d and Table 1). Interestingly, abnormal fibrous tissue and adipose tissue also expressed these mTOR pathway components (Fig. 3e-l and Table 1). Previous studies demonstrated that p-S6K1 was not detected in non-neoplastic fibrous tissue and adipose tissue [16, 17], besides p-S6K1 showed sporadic expression in normal vessels [18, 19]. Furthermore, both p-AKT and p-4EBP1 were not detected in non-neoplastic vessels, fibrous tissue and adipose tissue [16, 17, 19–21]. Thus, PI3K/AKT/mTOR pathway in mesenchymal malformation of FAVA is activated compared to that in normal tissue. These observations were consistent with the previous reports that *PIK3CA* mutations have been detected in adipocytes in PROS [22], subcultured fibroblasts in FAVA [6, 7] and lymphatic endothelial cells in LM [7, 23]. Moreover, *PIK3CA* mutation in fibroblast cell line excessively promoted PI3K/AKT/mTOR signaling and phosphorylation of its downstream [6, 24]. Hence, the phosphorylation of S6K1 and 4EBP1 may promote not only angiogenesis and lymphangiogenesis, but also fibrogenesis and adipogenesis in FAVA.

Jonathan E et al. (2017) presented two cases of FAVA with mTOR inhibitor sirolimus treatment [3]. Before that, there were no specific treatment for FAVA because of the overall low incidence. A few patients underwent surgical excision, sclerotherapy and cryablation [2]. Sirolimus has been shown to improve various vascular malformations by suppressing activation of mTOR, S6K1 and 4EBP1 [25]. In fact, sirolimus was rapidly effective for FAVA patients [3]. However, how sirolimus affected FAVA remains unclear. Our results suggested that sirolimus suppressed phosphorylation of mTOR pathway in not only abnormal vessels but also fibro-adipose tissue in FAVA.

## Conclusions

FAVA is a rare vascular malformation with *PIK3CA* mutation. We presented three new cases of FAVA. Histological and immunohistochemical analyses revealed that LVM was an important finding in FAVA, and that the mTOR pathway components were expressed in abnormal fibrous tissue, adipose tissue and vascular malformation.

## Data Availability

The surgical materials and the datasets analyzed during the current study are available from the corresponding author on reasonable request.

## Abbreviations

4EBP1: Eukaryotic translation initiation factor 4E-binding protein 1
FAVA: Fibro-adipose vascular anomaly
LM: Lymphatic malformation
LVM: Lymphatic venous malformation
mTOR: Mammalian target of rapamycin
PIK3CA: Phosphatidylinositol-4, 5-bisphosphate 3-kinase catalytic subunit alpha
PROS: The spectrum of PIK3CA-related overgrowth syndromes
S6K1: Ribosomal protein S6 kinase 1
VM: Venous malformation
